# Homeostatic pockets of interferon lambda-stimulated gene production in the intestine are associated with localized exposure to bacterial microbiota

**DOI:** 10.1080/19490976.2024.2447830

**Published:** 2024-12-30

**Authors:** David A. Constant, Jacob A. Van Winkle, Kimberly A. Meyer, Shelby R. Madden, Bryan Ramirez Reyes, Timothy J. Nice

**Affiliations:** Department of Molecular Microbiology and Immunology, Oregon Health & Science University, Portland, Oregon, USA

**Keywords:** Interferon lambda, innate immunity, intestinal epithelium

## Abstract

Tolerance of enteric microbiota and clearance of potential pathogens is critical for gut homeostasis. We previously found that the healthy small intestine of mice contains discrete pockets of antiviral gene expression that depend on bacterial microbiota colonization and interferon lambda (IFN-λ) receptor expression by intestinal epithelial cells. We now use spatial transcriptomic profiling of homeostatic interferon response pockets to show that they contain a broader cytokine signature that is consistent with activation of innate immune sensors, including toll-like receptors (TLRs). Additionally, we find that these homeostatic innate immune responses are associated with increased local abundance of luminal bacteria, but not with particular bacterial species or overt defects in the epithelial barrier. We find that deficiency of the TLR adaptor MyD88 in hematopoietic cells significantly reduces the abundance of homeostatic innate immune pockets, and stimulation with the bacterial product lipopolysaccharide preferentially stimulates IFN-λ production by intestinal immune cells. We propose that localized increases in proximity of bacterial microbiota to intestinal epithelium result in increased homeostatic uptake of TLR ligands, which stimulates resident immune cells to produce cytokines, including IFN-λ. These localized innate immune responses enhance the immunological barrier, maintaining homeostasis with beneficial microbes and protecting against potential occult pathogens that may be present among the luminal microflora.

## Introduction

The intestinal epithelium must perform multiple and often contradictory functions: absorbing nutrients and secreting waste, while also tolerating bacterial microbiota and controlling invading pathogens. Interferon lambda (IFN-λ) is an antiviral cytokine that primarily stimulates epithelial cells and is both necessary and sufficient for control of many enteric pathogens.^1^ Previously, we have shown that murine bacterial microbiota stimulates a highly localized homeostatic IFN-λ response that provides preemptive protection from infection with murine rotavirus (mRV) independent of type I IFN signaling.^2^ The homeostatic interferon-stimulated gene (ISG) expression pattern we observed in this previously published work was restricted to individual villi or small groups of villi near one another and was ablated by knockout of the IFN-λ receptor or antibiotics treatment to deplete the bacterial microbiota. These intriguing findings prompted the studies presented here, in which we investigate the broader gene expression signature associated with the localized homeostatic IFN-λ response, the spatial relationship of this response to bacteria, and the role of toll-like receptor signaling in regulating this response.

IFN-λ production is stimulated by pattern recognition receptor (PRR) activation. Toll-like receptors (TLRs) are important PRRs which stimulate production of interferons and other cytokines via adaptor proteins such as myeloid differentiation primary response protein 88 (MyD88) and TIR domain containing adaptor molecule 1 (TICAM1, also known as TRIF). In our prior studies, we have shown that oral exposure to the bacterial product lipopolysaccharide (LPS) is partially sufficient for stimulating localized homeostatic IFN-λ response.^2^ However, the specific role of bacterial microbiota and the broader transcriptional character of localized homeostatic responses remained unknown. In our work described here, we use spatial transcriptomic and imaging approaches to define the localized immune response and its association with bacteria. Additionally, we demonstrate that functional MyD88 signaling in hematopoietic cells is required for an intact homeostatic IFN-λ response.

## Results and discussion

### Homeostatic interferon lambda responses are accompanied by a broad inflammatory signature

**Figure 1.**
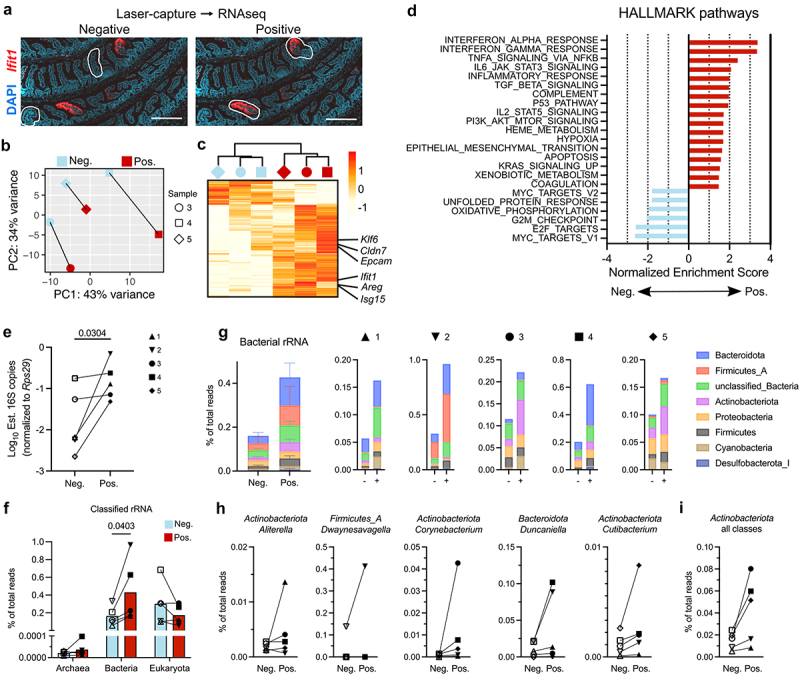
Discrete pockets of homeostatic interferon lambda response correlate with broader inflammatory signature and increased presence of bacterial microbiota. Laser-capture microdissection (LCM) was used to isolate paired *Ifit1*-negative regions and *Ifit1*-positive regions from mouse small intestinal tissue sections. (a) Representative images of two neg. (left) and pos. (right) regions captured by LCM. b–i. Paired neg. And pos. captures from five mice were prepared for sequencing as indicated in methods. Two mice were removed from mouse transcriptome analysis (b–d) due to high contamination of genomic DNA, but were included in rRNA analysis (e–i). (b) Principal component analysis of transcript counts. (c) Heatmap of transcript counts (scaled by row) for genes significantly different between neg. and pos. captures (padj <0.05). (d) HALLMARK pathways enriched in *Ifit1*-negative (blue bars, negative values) or -positive (red bars, positive values). (e) Quantitative RT-PCR measurements of bacterial 16S rRNA transcripts from extracted LCM RNA. f–i. Unmapped reads were mined for ribosomal RNA (rRNA) sequences and classified against the genome taxonomy database. (f) Proportions of total reads classified as rRNA from the indicated domains of life. (g) Bacterial rRNA classifications broken down by phylum. Proportions of total reads shown as average across all mice (left panel) or for each individual mouse as indicated by symbols above graphs. (h) Different genera with the greatest fold-change in *Ifit1*-positive regions for each mouse. (i) Phylum with the most consistent increase in *Ifit1*-positive regions across all mice. Statistical significance was tested for in (e) by one-tailed paired t-test and in (f) by two-way ANOVA with Sidak multiple comparisons test. Symbol shapes for each mouse are matched across all data panels.

A major outstanding question from our prior observations of homeostatic interferon-stimulated gene (ISG) expression is what defines their localized occurrence. We therefore performed laser-capture microdissection (LCM) of intestinal regions that were either negative or positive for ISG (*Ifit1*) expression in wild-type mouse small intestines (Figure 1a). We isolated RNA from paired *Ifit1-*negative and -positive regions from individual mice and performed RNA-seq. Gene expression analysis of three mice revealed that paired *Ifit1*-negative and -positive regions were separated similarly along axes of a principal component analysis (PCA) (Figure 1b). This suggested broad similarity in the transcriptional signature of homeostatic ISG regions. Indeed, differential gene expression analysis identified 201 genes significantly (padj <0.05) upregulated and 54 genes significantly downregulated in *Ifit1-*positive regions relative to paired negative regions (Figure 1c, supplementary table 1a). *Ifit1* was enriched ~ 35-fold in positive regions, confirming the successful capture of homeostatic pockets.

To evaluate the transcriptional changes between *Ifit1-*negative and -positive regions, we performed gene set enrichment analysis (GSEA) of HALLMARK pathways. The most significantly enriched HALLMARK pathways were IFN responses (Figure 1d, supplementary table 1b), indicating that ISGs are broadly co-enriched within *Ifit1-*positive regions. In addition to ISGs, we observed enrichment of HALLMARK pathways for other cytokine responses including tumor necrosis factor alpha (TNFα), interleukin 6 (IL-6), and transforming growth factor beta (TGF-β) (Figure 1d). Notably, these upregulated signatures included genes associated with mucosal healing and increased barrier integrity (*Areg*, *Klf6*, *Cldn7*, *Epcam*). In contrast, HALLMARK pathways for proliferation signatures (E2F and MYC targets) were downregulated in *Ifit1*-positive regions, suggesting that reduced proliferation accompanies the homeostatic ISG response (Figure 1d, bottom). These results demonstrate that ISG-positive regions are undergoing active immune responses that involve cytokines beyond IFN, including signatures of mucosal healing.

### Homeostatic interferon lambda responses correlate with local abundance of bacterial microbiota

Our prior study indicated that localized pockets of ISG expression in the intestine are dependent on bacterial microbiota,^2^ but the localization of bacteria relative to immune pockets was unknown. To analyze association between microbes and *Ifit1*-positive regions captured by LCM, we first quantified bacterial 16S transcripts in RNA extracted from these samples by RT-qPCR and observed greater abundance in all *Ifit1*-positive versus negative samples (Figure 1e). We then identified ribosomal RNA (rRNA) present within unmapped LCM reads and classified rRNA taxonomy based on the genome taxonomy database (Figure 1f–i, supplementary table 1c). A small proportion of total reads (0.1–1%) were classified as rRNA from *Archaea*, *Bacteria*, or *Eukaryota* domains (Figure 1f). Archaea rRNA sequences were very infrequent (<0.0001%) and not analyzed further. Eukaryotic rRNA sequences were not significantly different between *Ifit1*-negative and -positive regions (Figure 1f), and their phylogenetic assignments indicated *Mus* and *Homo* contaminant, or likely dietary components (i.e. *Zea*) (supplementary table 1d). Therefore, *Eukaryota* rRNA served as an internal control. In contrast to *Eukaryota*, *Bacteria* rRNA sequences were significantly more abundant in *Ifit1*-positive regions relative to paired *Ifit1*-negative regions from each of five mice (Figure 1f, supplementary table 1d). The taxonomic assignment of bacterial rRNA reads was consistent with microbiota known to be present in the mouse intestine, including *Bacteroidota* and *Firmicutes* phyla (Figure 1g, supplementary table 1d). The specific composition of bacteria was different between replicate mice, but there was consistently greater abundance of bacteria in *Ifit1*-positive regions within each mouse (Figure 1g). The genera with the greatest fold-increase in *Ifit1*-positive regions was different for each mouse (Figure 1h), with mouse #2 having a substantial fraction of reads coming from segmented filamentous bacteria (*Dwaynesavagella*) and mouse #4 having a high fraction of reads coming from *Duncaniella*. The most consistently increased phylum was *Actinobacteriota*, which was higher in *Ifit1*-positive regions from all mice (Figure 1i). Taken together, these data indicate that local abundance of diverse taxa within the bacterial microbiota correlates with a localized innate immune response.

**Figure 2.**
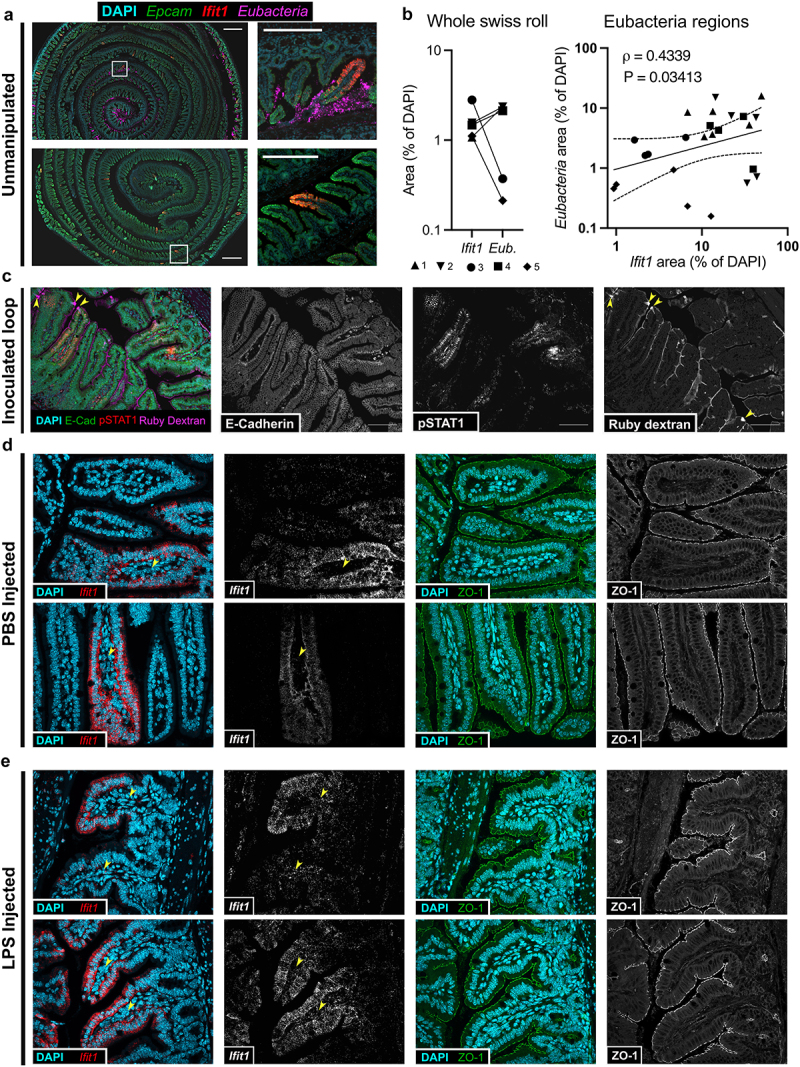
Discrete pockets of homeostatic interferon lambda response correlate with bacterial abundance. (a) Representative images of serial sections from LCM Swiss rolls stained by RNAscope for *Epcam*, *Ifit1*, and *Eubacteria*. Scale bars 500 µm or 200 µm (inset). (b) *Ifit1* area and Eubacteria area within entire Swiss roll images (left) or selected Eubacteria regions (right), normalized to DAPI area. Eubacteria regions were selected with investigator blinded to *Ifit1* signal. Entire Swiss roll images and selected regions are shown in Supplementary figure 1. Symbol shapes represent different mice from figure 1. (c) Representative image of small intestine segments inoculated with ruby dextran to visualize barrier integrity and co-stained with phospho-STAT1, E-cadherin, and DAPI. Arrowheads indicate cells that have taken up ruby dextran at homeostasis. Scale bar 100 µm. (d,e) Representative 40X images of serially sectioned small intestine (ileum) from mice treated with 0.5 mL PBS (d) or 50 mg LPS in 0.5 mL PBS (e) Tissues were collected 6 hours after injection with PBS or LPS. Expression of *Ifit1* was visualized by RNAscope with DAPI counterstaining (left two panels). Using serial sections of the same tissues localization of ZO-1 was visualized by immunohistochemistry (right two panels). Local regions were selected manually for congruence across serial sections. Arrowheads indicate lamina propria regions of villi.

Bacteria within the small intestine are less dense than the colon, and small intestinal mucus is loosely organized. Bacteria can be found within this loose mucus layer, though they appear to rarely make direct contact with the epithelium.^3^ To assess whether there was visual evidence of a relationship between ISG expression and bacterial abundance, we stained for *Ifit1* and eubacteria nucleic acids in serial sections of the same Swiss rolls used for LCM (Figure 2a). We readily detected *Eubacteria* 16s rRNA in luminal regions near pockets of ISG (*ifit1*) signal but observed minimal bacterial signal within epithelial or sub-epithelial regions (Figure 2a, Supplementary figure S1). These data suggest that ISG expression is not triggered by overt bacterial invasion. There was substantial variance in Eubacteria signal between mice and no correlation between cumulative Eubacteria area and *Ifit1* area within entire Swiss rolls (Figure 2b). However, large areas of tissue had no visible Eubacteria, suggesting that they were lost during tissue preparation or below the limit of detection by fluorescence imaging. Therefore, to examine regional correlations between bacterial abundance and homeostatic *Ifit1*, we blindly selected regions that contained visible *Eubacteria* from each of five mice (Supplementary figure S1) and quantified the area of *Ifit1* fluorescence. We observed a significant positive correlation between Eubacteria area and *Ifit1* expression across regions (Figure 2b). These imaging data synergize with our LCM data (Figure 1e–i) to support the conclusion that there is increased bacterial abundance near regions with the greatest homeostatic response.

### Homeostatic pockets of interferon lambda response are not correlated with loss of barrier integrity

We next asked whether there was overt damage to the epithelial barrier surrounding homeostatic pockets. To directly visualize the integrity of the intestinal epithelial barrier, we inoculated segments of intestine with fixable ruby dextran solution. This assay uses a fluorescently conjugated membrane-impermeable compound to label the lumen of the intestine.^4^ We observed coating of the epithelial surface with ruby dextran, and uptake of ruby dextran into what are likely goblet cell-associated passages (GAPs; Figure 2c), which are sites of homeostatic antigen sampling.^5^ We co-stained for E-cadherin to mark epithelial junctions and phospho-STAT1 to mark homeostatic IFN responses and found that STAT1-positive pockets excluded ruby dextran and retained junctional E-cadherin staining (Figure 2c).

In a separate experiment, we injected mice intraperitoneally with LPS to bypass the epithelial barrier and stimulate widespread innate immune responses (Figure 2d–e, Supplementary figure S1F). In PBS-injected control mice, *Ifit1* expression was localized to pockets as expected (Figure 2d), but *Ifit1* expression was ubiquitous in LPS-injected mice (Figure 2e). Quantitation of ISG signal in PBS vs. LPS injected mice confirmed that *Ifit1* and *Isg15* expression was consistently and strongly upregulated upon LPS stimulation (supplementary Fig. S1F). The LPS-stimulated *Ifit1* expression was notably strong in the epithelium but was also present in the lamina propria (Figure 2e, arrowheads), suggesting heterogeneous IFN responses. We stained serial intestinal sections for the tight junction protein Zonula Occludens 1 (ZO-1) as a measure of tight junction localization and abundance. We observed no evidence of tight junction breakdown in either PBS- or LPS-injected mice (Figure 2d,e). These data suggest that there is no overt damage associated with epithelial IFN responses, but do not rule out the possibility that damage is temporally or spatially undetectable by our methods.

Taken together with our sequencing data, these imaging results are consistent with our hypothesis that localized ISG responses involve homeostatic sampling of products from nearby bacterial microbiota, in the absence of barrier breach or active invasion of the epithelium by microbes.

### Myd88 signaling in hematopoietic cells promotes homeostatic interferon lambda response.

**Figure 3.**
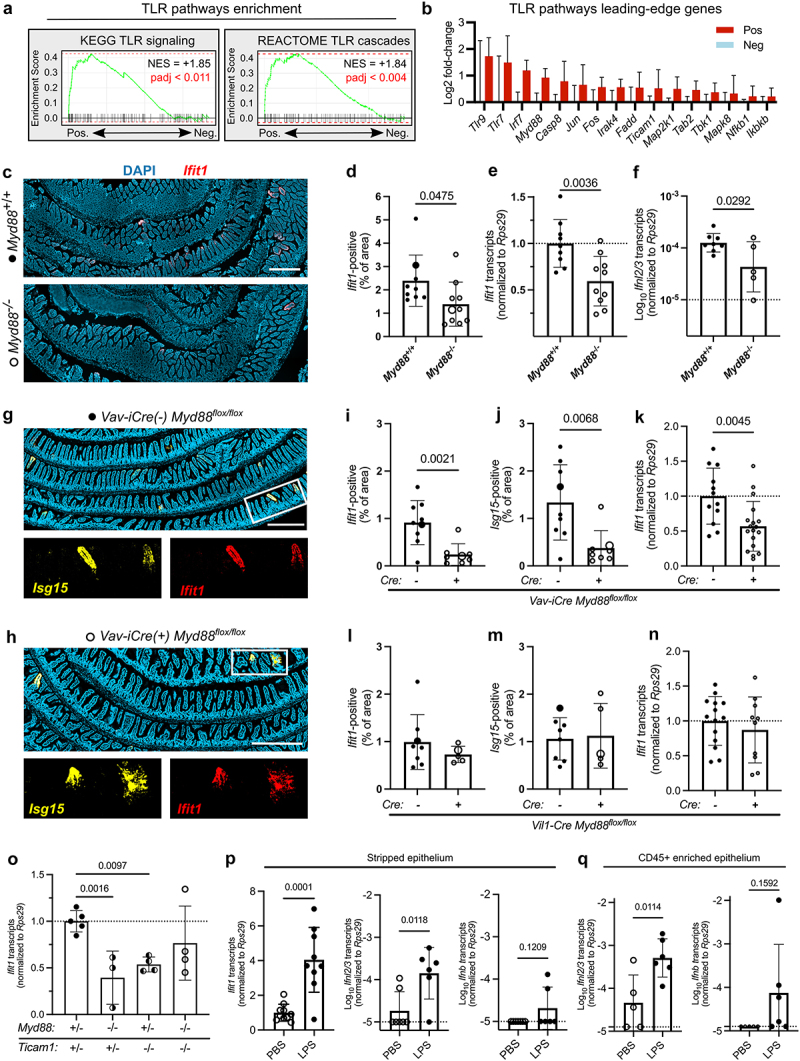
Myd88 signaling in hematopoietic cells promotes the homeostatic interferon lambda response. (a) TLR pathway gene set enrichment analysis (GSEA) from LCM data using KEGG (left) and REACTOME (right) databases. (b) Fold-change in expression of leading-edge genes from TLR cascade GSEA plots for *Ifit1*-positive (red) regions relative to *Ifit1*-negative (blue) regions. (c) Representative images of Swiss rolls from WT and *Myd88*^*-/-*^ mice showing DAPI (blue) and *Ifit1* (red) signal. (d) Quantified *Ifit1*-positive area normalized to DAPI from *n* = 9 WT and *n* = 10 *Myd88*^*-/-*^ mice. Large symbols indicate values for the representative images shown in (c). (e) *Ifit1* transcripts detected by qRT-pcr in stripped epithelium from WT and *Myd88*^*-/-*^ mice. (f) *Ifnl2/3* transcripts detected by qRT-pcr in CD45-enriched cells from WT and *Myd88*^*-/-*^ epithelial strips. (g, h) Representative images of Swiss rolls from *vav-iCre(+) Myd88*^*fl/fl*^ (g) and *vav-iCre(-) Myd88*^*fl/fl*^ mice (h) showing DAPI, *Ifit1*, and *Isg15* (yellow) signal. (i, j) Quantified *Ifit1*-positive (i) and *Isg15-*positive (j) area normalized to DAPI from *n* = 9 *vav-iCre(-)* and *n* = 8 *vav-iCre(+)* mice. Large symbols indicate values for the representative images shown in (g,h). k. *Ifit1* transcripts in stripped epithelial cells from *vav-iCre Myd88*^*fl/fl*^ mice. (l, m) Quantified *Ifit1*-positive (l) and *Isg15-*positive (m) area normalized to DAPI from *n* = 8 *Vil1-cre(-)* and *n* = 5 *Vil1-Cre(+)* mice. Large symbols indicate values for the representative images shown in supplemental figure 2. n–p. Transcripts of *Ifit1, Ifnl2/3*, and *ifnb* as indicated in graphs in stripped epithelial cells from *Vil1-cre Myd88*^*fl/fl*^ mice (n), mice with indicated *Myd88/Ticam1* genotypes (o), and mice injected intraperitoneally with 50 mg LPS or PBS. (p, q) *Ifit1* (p), *Ifnl2/3* (p, q), and *ifnb* (p, q) transcripts in stripped epithelium (p) or CD45-enriched cells from epithelial strips (q) of mice injected intraperitoneally with LPS. Statistical significance by unpaired t-test (d–f, i–k, l–n, p,q) or one-way ANOVA with Sidak multiple comparisons test (o).

The enrichment of IFN, IL-6, and TNF pathway genes in homeostatic pockets suggested a localized activation of TLRs. Indeed, further GSEA analyses of LCM data revealed enrichment of toll-like receptor (TLR) response genes curated by KEGG and REACTOME in *Ifit1*-positive compared to *Ifit1*-negative regions (Figure 3a). The leading-edge genes that were in common between these KEGG and REACTOME pathways include TLRs themselves, the *Myd88* adaptor gene, MAP kinase genes, and NFκB genes (Figure 3b). To quantify the contribution of Myd88 to localized IFN-λ responses within the intestinal epithelium, we performed *Ifit1* RNAscope in intestinal tissues of littermate *Myd88*^*+/+*^ and *Myd88*^*-/-*^ mice. *Ifit1*-positive regions of epithelium were significantly less abundant in *Myd88*^*-/-*^ mice in total DAPI-normalized area, and the total number of observed pockets was also reduced (Figure 3c–d, supplementary figure S2). Parallel measurements of *Ifit1* transcripts in stripped epithelial fractions by qPCR corroborated a *Myd88*-dependent contribution to *Ifit1* expression within ileal epithelium (Figure 3e). We previously found that homeostatic *Ifnl2/3* transcripts are found predominantly within CD45-positive cells.^2^ We measured *Ifnl2/3* transcripts within enriched CD45-positive cells from the stripped epithelia of *Myd88*^*-/-*^ mice and observed a reduction relative to littermate *Myd88*^*+/+*^ controls (Figure 3f). These data indicate that Myd88 promotes IFN-λ production by epithelium-associated immune cells, and local ISG production by the epithelium at homeostasis. In contrast, during norovirus infection or after poly(I:C) injection, IFN-λ is secreted from IECs,^6,7^ highlighting that homeostatic IFN-λ signaling is regulated in a distinct manner.

We hypothesized that immune cells require cell-intrinsic *Myd88* to produce IFN-λ and stimulate epithelial ISG responses. To test this, we bred *Myd88*^*flox/flox*^ mice with *Vav*^*iCre*^ or *Vil1*^*Cre*^ mice to generate conditional *Myd88* knockouts specific for hematopoietic cells and intestinal epithelial cells, respectively. We collected intestinal tissues from these mice and stained them for *Ifit1* and co-stained for another ISG (*Isg15*). Villi expressing *Ifit1* generally also express *Isg15*, indicating a coordinated ISG response at these locations (Figure 3g–h). We observed an approximately 50% reduction in *Ifit1-* and *Isg15*-positive area (Figure 3i–j) in *Vav*^*iCre+*^*Myd88*^*flox/flox*^ mice relative to *Vav*^*iCre-*^*Myd88*^*flox/flox*^ mice as well as a similar reduction in the number of ISG-positive villi (supplementary figure S2), and a similar reduction in *Ifit1* signal by qPCR from stripped epithelium (Figure 3k). These data suggest TLR stimulation in hematopoietic cells is partly required for homeostatic expression of *Ifit1* in the intestine. By contrast, there was no reduction in *Ifit1* or *Isg15* signal by imaging or qPCR from *Vil1*^*Cre+*^*Myd88*^*flox/flox*^ mice relative to *Vil1*^*Cre-*^*Myd88*^*flox/flox*^ mice (Figure 3l–n, supplementary figure S2), demonstrating that TLR stimulation in IECs is dispensable for homeostatic expression of *Ifit1* or *Isg15*.

Because we still observed some homeostatic *Ifit1* transcripts in both full-body and hematopoietic-specific *Myd88*^−/−^ mice, we hypothesized that the other main TLR signaling adaptor, TRIF, might be contributing to homeostatic IFN-λ production. To test this, we bred *Myd88*^*-/-*^ and *Ticam1*^−*/-*^ (TRIF gene) mice to generate doubly-heterozygous F1 offspring, which were then intercrossed to generate littermate F2 offspring with either heterozygous or homozygous knockout for each of these TLR signaling adaptor genes. *Ifit1* transcript levels in ileal epithelium were reduced approximately 50% in *Myd88* or *Ticam1* knockouts relative to double-heterozygotes, but were not further reduced in double-knockouts (Figure 3o). These data suggest redundant contributions of both *Myd88* and *Ticam1* to homeostatic ISG expression in the small intestine. The remaining 50% ISG signal present in the epithelium of *Myd88*/*Ticam1* double knockout mice suggests that additional innate sensors non-redundantly contribute to localized IFN-λ responses.

Our imaging in Figure 2e of LPS injected mice indicated predominant epithelial expression of *Ifit1*, suggesting involvement of IFN-λ. Therefore, we sought to test whether systemic TLR stimulation by LPS injection further amplified IFN-λ production by intestinal immune cells and epithelial expression of ISGs. Four hours after exposure, we observed a roughly four-fold increase in *Ifit1* within the stripped epithelium by qPCR (Figure 3p, left), supporting our observations by imaging (Figure 2e). We also detected increased levels of *Ifnl2/3* transcripts, but little to no expression of a type I IFN gene (*Ifnb*) (Figure 3p, right panels) in total stripped epithelium. We enriched CD45-positive immune cells from the epithelial fraction to quantify their contribution to the LPS-stimulated production of IFNs. Transcripts for a type I IFN gene (*Ifnb*) were inconsistently detected, and not significantly increased (Figure 3q, right). In contrast, *Ifnl2/3* transcripts were enriched ~ 10-fold as shown by qPCR (Figure 3q, left). These data suggest preferential production of IFN-λ by a hematopoietic cell type in the intestine that can be amplified by systemic LPS exposure. Together, these data support a model whereby transient, localized increases in bacterial abundance within the intestinal lumen result in regionally increased homeostatic sampling of stimuli for TLRs and other PRRs.

Our results are based on static ‘snapshots’ of events occurring in the intestine, limiting our temporal understanding of the dynamic molecular events leading to localized homeostatic responses. Nonetheless, our data suggests a model whereby increased regional sampling drives production of IFN-λ and other cytokines to elicit a local preemptive innate immune response. We propose that this mechanism may restrict homeostatic innate immune activity to sites with the greatest potential for pathogen exposure.

## Conclusion

IFN-λ has well-described roles in restricting viral infection and promoting wound healing in the intestine.^6–11^ The role of this cytokine in maintaining homeostasis is much less well understood. In the absence of infection or injury, IFN-λ is present in the intestine at low levels and may provide preemptive protection from viral infections.^2^ Our findings here indicate that there is a TLR-mediated stimulatory mechanism for secretion of homeostatic IFN-λ that is spatially correlated with bacterial abundance. Although our sequencing results indicated that many bacterial species can be associated with homeostatic pockets, it is possible that specific bacterial species with adhesive or mucus-degrading properties might provide stronger stimulation. Additionally, the specific immune cell type responsible for producing homeostatic IFN-λ remains to be determined. It is notable that a source of IFN-λ at homeostasis is an immune cell, which is different than the predominantly enterocyte-derived IFN-λ reported during norovirus infection.^6^ In sum, our results advance understanding of homeostatic IFN-λ responses by defining a broader inflammatory program, correlating them with local bacterial abundance, and implicating TLR stimulation of immune cells.

## Materials and methods

### Mice

C57BL/6J (wild-type) mice, *Myd88*^*-/-*^ (B6.129P2(SJL)-*Myd88*^*tm1.1Defr*^/J, stock #009088), *Ticam1*^*-/-*^ (C57BL/6J-*Ticam1*^*Lps2*^/J, stock #005037), *Myd88*^*flox*^ (B6.129P2(SJL)-*Myd88*^*tm1Defr*^/J, stock #008888), *Vav-iCre* (B6.Cg-*Commd10*^*Tg(Vav1-icre)A2Kio*^/J, stock #008610), *Vil-cre* (B6.Cg-Tg(Vil1-cre)1000Gum/J, stock #021504) mice were originally purchased from Jackson Laboratories and bred in a specific pathogen-free facility at Oregon Health & Science University. All experiments used littermate control and experimental mice. Animal protocols were approved by the Institutional Animal Care and Use Committee at OHSU (protocol #IP00000228) in accordance with standards provided in the Animal Welfare Act.

### RNAscope

Intestinal tissue Swiss rolls were fixed in 10% neutral-buffered formalin (NBF) for 18–24 hr and embedded in paraffin. Tissue sections (5 μm) were cut and stored at room temperature with desiccant until staining. RNA *in situ* hybridization was performed using the RNAscope Multiplex Fluorescent v2 kit (Advanced Cell Diagnostics, Newark, CA) per protocol. As indicated in figure legends, slides were stained with anti-sense probes for *Ifit1* (ACD, #500071), with or without *Epcam* (ACD, #418151), *Eubacteria* (#EB-16s-rRNA), and *Isg15* (#559271). Slides were counterstained with DAPI, mounted with ProLong Gold antifade reagent (ThermoFisher), and imaged using a Zeiss ApoTome2 on an Axio Imager, with a Zeiss AxioCam 506 (Zeiss). Collected images were batch processed in Zeiss Zen 3.1 using unstained control slides to set background values. Fluorescent signal was quantified as positive fluorescent area for each target relative to the total fluorescent area of the tissue section as determined by DAPI. For *Eubacteria* correlation analyses, regions of interest (ROI) containing varying amounts of visible *Eubacteria* were chosen in Zen image analysis software blinded to *Ifit1* signal. From each Swiss roll, 4–6 ROIs (5×10^5^ to 6 × 10^6^ pixels^2^) were selected for further analysis. In ImageJ, Single-channel images of DAPI, Epcam, Ifit1, and 16S rRNA (eubacteria) were converted to 8-bit format and brightness thresholding was applied.

### Laser-capture microdissection and RNA sequencing

ROIs were selected by RNAscope staining of tissue sections for *Ifit1*. Captures were isolated on an Arcturus LCM instrument from unstained serial sections mounted on RNAse free membrane slides (Molecular Machines & Industries, #50102) using CapSure Macro LCM caps (ThermoFisher Scientific, #LCM0211). RNA was isolated from ROIs using a Quick-RNA FFPE Miniprep (Zymo Research, cat#R1008) kit and checked for quality on a Bioanalyzer (Agilent). RNA was prepared for sequencing with a SMART-Seq Stranded Kit (Takara Bio, cat#634442) using ribosomal RNA depletion and libraries were sequenced on an Illumina instrument at the OHSU Massively Parallel Sequencing Shared Resource.

### Immunohistochemistry

Paraffin-embedded tissue sections were prepared for immunohistochemistry as for RNAscope. For ruby dextran permeability assay, ileum tissue was dissected and filled with 2 mL of fixable ruby dextran (250 μg/mL) and incubated for 1.5–2 min prior or to fixation and processing. Antigen retrieval was performed in 0.1 M sodium citrate solution, pH 6, for 15 min at 95°C (ruby dextran) or in 10 mm Tris, 1 mm EDTA, pH 9.0 solution for 45 min at 95°C (ZO-1). The following primary antibodies were used to stain markers indicated in figure legends: anti-E-cadherin (1:200, Clone 36, Beckton Dickson #610182), anti-phospho-STAT1 (Y701) (1:400, clone 58D6, Cell Signalling Technology #9167), anti-ZO-1 (1:200, ThermoFisher PA5–85256). Species-specific Alexa Fluor-conjugated secondary antibodies (ThermoFisher) were used for fluorescent microscopy, performed as for RNAscope.

### RNAseq analysis

Adaptor-trimmed reads were aligned to the mouse genome (GRCm39) using STAR version 2.7.9a, and mapping quality was evaluated using RSeQC and MultiQC. All samples had between 40 million and 51 million uniquely mapped reads with uniform gene body coverage. Samples from two mice had greater than 20% of reads distributed among ‘Other_intergenic’ features, suggesting high genomic DNA contamination, so these mice were excluded from mouse transcriptome analysis and used only for ribosomal sequence analysis. Read counts per gene transcript were determined using the featureCounts program, and differential expression analysis was performed using DEseq2, with a multi-factor design to control for variance between mice (design = ~ Capture + Sample). PCA and heatmaps were generated using regularized logarithm (rlog)-transformed data. The table of differential gene expression from DEseq2 was ordered using the Wald statistic to generate a pre-ranked list of genes for analysis using the fast gene set enrichment (fgsea) R package. Reads that were unmapped to mouse genome were mined for ribosomal RNA (rRNA) sequences using Ribodetector,^12^ using the ‘–ensure rrna’ setting to limit hits to high-confidence assignments. High-confidence rRNA reads from Ribodetector were subsequently classified with lenient cutoffs (threshold = 40%) using IdTaxa^13^ against the Genome Taxonomy Database (GTDB) release 207. RNA sequencing data obtained in this study have been deposited in the NCBI Gene Expression Omnibus under GEO series accession number GSE269701.

### Epithelial strip and magnetic bead enrichment

Epithelial fractions were prepared by non-enzymatic dissociation as previously described.^2^ Briefly, mouse ileum was longitudinally opened and agitated by shaking in stripping buffer (5% fetal bovine serum, 5 mm EDTA, 1 mm dithiothreitol, 1X penicillin-streptomycin-L-glutamine (Gibco), in PBS) for 20 min at 37°C. Epithelial stripping solution was filtered through a 70 μm cell strainer and dissociated cells were collected for use in qPCR analysis, flow cytometry, and magnetic bead enrichment. For magnetic enrichment, Dissociated IECs were collected and immune cells were enriched after flow cytometry staining for anti-CD45-APC (clone 30-F11; BioLegend) using MojoSort Mouse anti-APC Nanobeads (BioLegend, #480072) according to manufacturer protocols.

### Quantitative PCR

RNA from stripped IECs was isolated with TRIzol (Life Technologies, Carlsbad, CA) per the manufacturer’s protocol. RNA from magnet-enriched cells was isolated with a Zymo Quick-RNA Viral Kit (Zymo Research, Irvine, CA). Isolated RNA was treated with the DNAfree kit (Life Technologies) and used as a template for cDNA synthesis (ImProm-II reverse transcriptase system, Promega, Madison, WI). Quantitative PCR was performed (PerfeCTa qPCR FastMix II, QuantaBio, Beverly, MA) and absolute transcript copy numbers were determined using standard curves generated from synthetic gBlocks (IDT) containing target sequences. Absolute copy numbers were normalized to gene copies of ribosomal protein S29 (*Rps29*). The following TaqMan assays for selected genes were obtained from IDT (Coralville, IA): *Rps29* (Mm.PT.58.21577577), *Ifit1* (Mm.PT.58.32674307). TaqMan assays for *Ifnl2/3* and *Ifnb1* were previously designed^10^ with the following sequences: *Ifnl2/3* (Primer 1 – GTTCTCCCAGACCTTCAGG, Primer 2 – CCTGGGACCTGAAGCAG, Probe – CCTTGCAGGCTGAGGTGGC); *Ifnb1* (Primer 1 – CTCCAGCTCCAAGAAAGGAC, Primer 2 – GCCCTGTAGGTGAGGTTGAT, Probe – CAGGAGCTCCTGGAGCAGCTGA).

## Supplementary Material

Supplemental Material

## Data Availability

The RNA sequencing data that support the findings of this study are openly available in the NCBI Gene Expression Omnibus under GEO series accession number GSE269701.
