# Supplementary figures and images for: Homeostatic pockets of interferon lambda-stimulated gene production in the intestine are associated with localized exposure to bacterial microbiota
Source: Gut Microbes. 2024 Dec 30;17(1):2447830. doi: 10.1080/19490976.2024.2447830 (PMC12269672; doi:10.1080/19490976.2024.2447830)

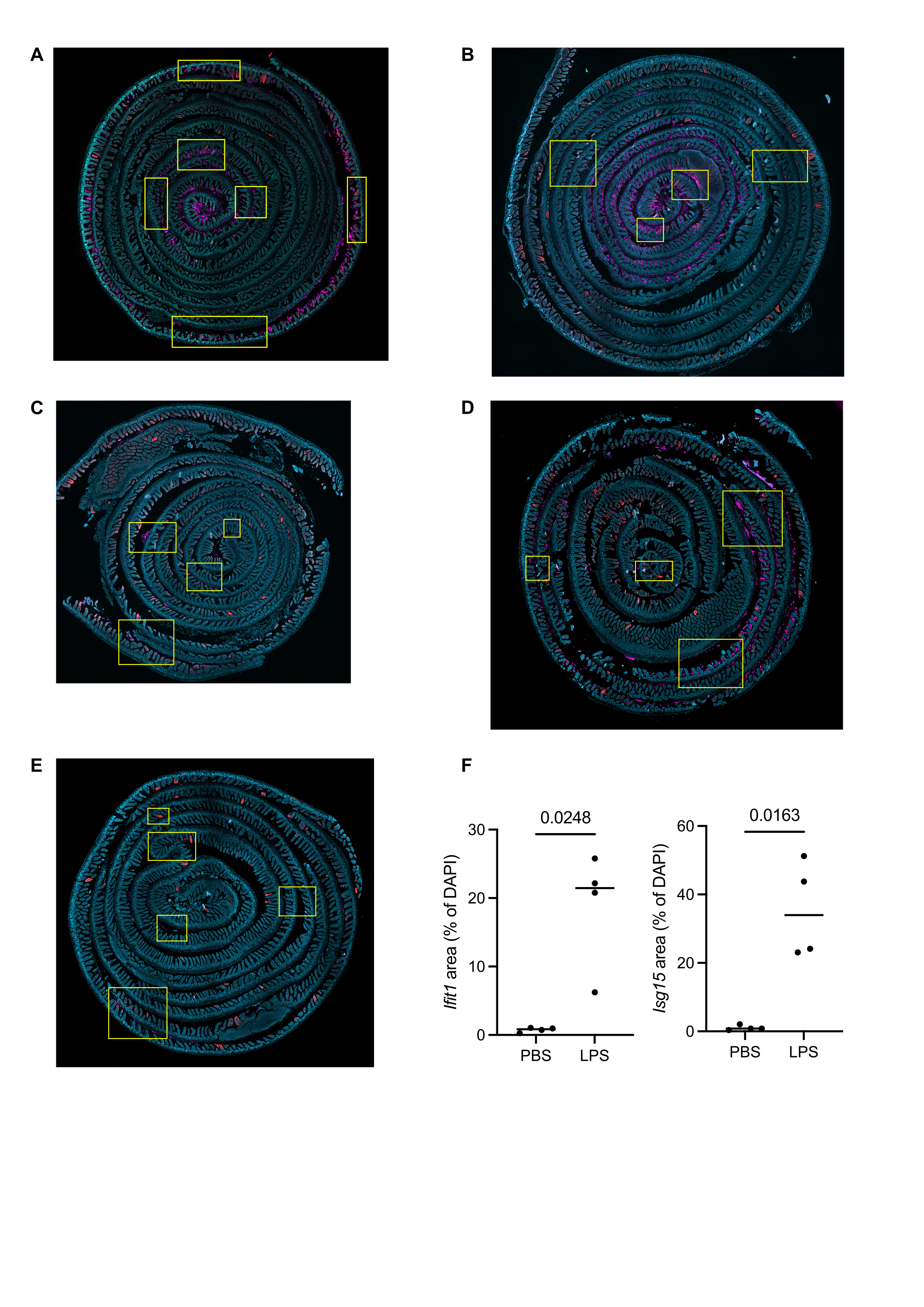

Supplement: Supplemental Material [file KGMI_A_2447830_SM6455.zip › kgmi-s-2024-1062-20241224163748/graphic/Supplementary Figure 1_revised.tiff]

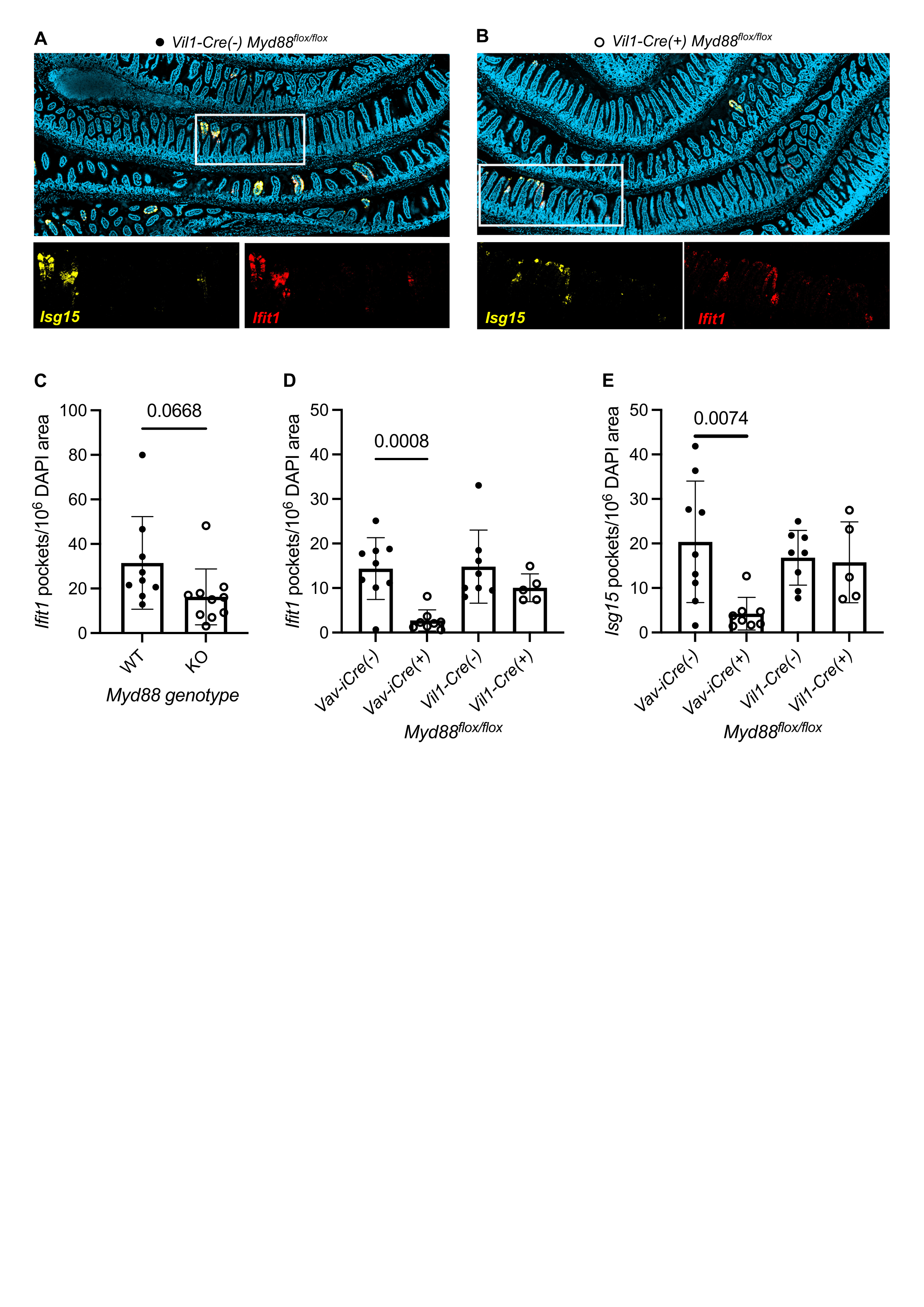

Supplement: Supplemental Material [file KGMI_A_2447830_SM6455.zip › kgmi-s-2024-1062-20241224163748/graphic/SupplementaryFigure2.tiff]
